# Emodin Suppresses Hyperglycemia-Induced Proliferation and Fibronectin Expression in Mesangial Cells via Inhibiting cFLIP

**DOI:** 10.1371/journal.pone.0093588

**Published:** 2014-04-01

**Authors:** Junjie Gao, Fangli Wang, Weisong Wang, Zhiguo Su, Canghui Guo, Shuyi Cao

**Affiliations:** Department of Nephrology, Cangzhou Central Hospital, Cangzhou, China; University of Alabama, Birmingham, United States of America

## Abstract

As one of the most serious microvascular complications of diabetes and a major cause of end stage renal disease, diabetic nephropathy (DN) is calling for effective treatment strategies. Here, we provide evidence that hyperglycemia can induce proliferation and decreasing apoptosis of mesangial cells (MCs) and subsequent renal dysfunction by up-regulating cellular FLICE-inhibitory protein (cFLIP). Treatment with emodin significantly turns down the accelerated cell cycle and proliferation of MCs cultured in high glucose (HG) via inhibiting cFLIP. *In vitro*, knockdown of cFLIP can arrest cell cycle and accelerate cell death by activating caspase-8, caspase-3 and caspase-9, and down-regulate proliferating cell nuclear antigen (PCNA). Our results also suggest that emodin regulates cFLIP expression in transcriptional level. Importantly, emodin lessens proteinuria and fibronectin expression in early-stage of streptozotocin (STZ)-induced diabetic rats. These findings demonstrate that emodin represent a promising strategy to prevent renal dysfunction in early-stage of diabetes mellitus.

## Introduction

Diabetes and hypertension are two major factors causing chronic kidney disease (CKD) [1], [2], [3]. Hyperglycemia plays a central role in the pathogenesis of diabetic nephropathy (DN), as shown by its action in promoting mesangial cells (MCs) proliferation and fibronectin expression in vitro [4], [5], [6]. DN is characterized early in its stage by glomerular hypertrophy and, especially, mesangial hypertrophy (caused by mesangial cell proliferation and extracellular matrix expression), which is associated with the eventual glomerulosclerosis and consequent deteriorating kidney function [7], [8], [9]. Fibronectin is one of the major ingredients of extracellular matrix (ECM) proteins synthesized by MCs. The excessive accumulation of fibronectin is often used as an index to evaluate the levels of matrix accumulation. Therefore, inhibiting both proliferation and fibronectin production in MCs is regarded as an effective strategy to ameliorate DN. Despite the great progresses have been made in the last decades, the mechanism involved in mesangial hypertrophy and fibronectin expression is not fully understood. More important, there is still no curative therapy for DN currently available. Therefore, effective agents for prevention and treatment of DN are in need.

emodin (1,3,8-trihydroxy-6-methylanthraquinone), a natural anthraquinone derivative isolated from the root and rhizome of *Rheum Palmatum*, is a Chinese herb widely used for immunosuppression, anti-in-flammation and anti-proliferation, despite that the mechanisms are not fully understood [10], [11]. Previous study demonstrated that emodin was effective to suppress cell proliferation and fibronectin expression in rat MCs cultured under high glucose (HG), however, some problems, such as the underlying mechanisms and if emodin is also available in vivo, are piled up. Based on previous findings, we hypothesized that emodin can exerts an inhibiting effect on HG induced proliferation and up-regulated expression of fibronectin in MCs.

Cellular FLICE-like inhibitor protein (cFLIP) is recognized as catalytically inactive homolog of caspase-8, which inhibits caspase-8 recruitment and processing at the death-inducing signaling complex (DISC) [12]. cFLIP encodes two splicing variants, short form (cFLIP_S_, contains two N-terminal death effector domains (DEDs)) and long form cFLIP (cFLIP_L_, contains two N-terminal DEDs and a C-terminal caspase-like domain that does not possess enzymatic activity) [13]. Overexpression of both long and short isoforms of cFLIP has been shown to protect against apoptosis mediated by death receptor including FasL and TRAIL in several cancer cells in vitro [14]. However, the effect of cFLIP on emodin-induced apoptosis, preventing proliferation and expression of fibronectin hasn't been disclosed.

## Methods and Materials

### Materials and reagents

Dulbecco's Modified Eagle's Medium (DMEM) and fetal bovine serum (FBS) was purchased form Gibco (Life technologies, Shanghai, China). An antibody that recognizes both the long and short isoforms of cFLIP (cFLIP_L_ and cFLIP_S_) (sc-5276) was purchased from santa cruz (Santa Cruz, USA). Another one against caspase-8 was from Alexis Biochemicals. Other antibodies against caspase-3 (ab90437), caspase-9 (ab25758), PCNA (ab2426) and fibronectin (ab2413) were from abcam (Cambridge, UK). Emodin, streptozotocin (STZ), D-glucose, mannitol, N-benzyloxycarbonyl-Val-Ala-Asp-fluoromethylketone (zVAD.fmk), bortezomib and other chemicals were purchased from Sigma (St. Louis, USA) unless otherwise stated.

### Cell culture, cFLIP overexpressing and knockdown

The rat glomerular MC line (HBZY-1) was from China Center for Type Culture Collection (Wuhan, China) and cultured in normal DMEM media (5.5 mM D-glucose) supplemented with 10% FBS in an atmosphere of 5% CO2 at 37°C. For HG media, additional D-glucose was supplemented to the normal DMEM media with a final D-glucose concentration at 25 mM. Phenotype of cultured MCs grown in HG environments resembles the phenotype of DN [15], [16], [17]. The osmotic control media was made by supplementing normal media with 19.5 mM mannitol. For overexpressing of cFLIP, cFLIP_L_ and cFLIP_S_ coding regions were PCR amplified and ligated into the pcDNA/V5-His TOPO vector according to the manufacturer's instructions (Life Technologies Inc.). MCs cells were cotransfected with each cFLIP expression construct and a construct expressing a puromycin resistance gene (pIRESpuro3, Clontech, BD Biosciences) for 48 h in DMEM with 10% FBS and 5.5 mM glucose. For knockdown, cells were infected with lentiviral vectors encoding short hairpin RNA (shRNA) for cFLIP or scrambled sequence. The rat cFLIPL shRNA vectors and scrambled negative control were purchased from GeneCopoeia (catalog no. RSH047914-HIVU6, clone ID NM_001033864.2). Both were placed into psiHIV-U6 vectors. Stably transfected MCs were selected and maintained in medium supplemented with 1 mg/ml puromycin (Life Technologies Inc.).

### Cell viability by 3-(4,5-dimethylthiazol-2-yl)-2,5-diphenyltetrazolium bromide assay and cell cycle flow cytometry

Rat glomerular MCs were seeded in 24-well plates at a density of 5,000 per well in medium containing 10% FBS with 5.5 mM D-glucose. Six replicate wells were used for each sample at each time point. At the indicated time point, medium was removed and serum-free medium containing 3-(4,5-dimethylthiazol-2-yl)-2,5-diphenyltetrazolium bromide (MTT; 0.5 mg/ml) was added into each well. Four hours after incubation at 37°C, cellular formazan product was dissolved with acidic isopropanol, and the absorbance at 570 nm was measured by spectrophotometry (Beckman Du640B). For the cell cycle flow cytometry assay, cells were digested by trypsin-EDTA, harvested as many as 1×10^6^ cells, and fixed in 70% ethanol at 4°C. After 12 h, cells were centrifuged (1,000×g, 5 min, 4°C), resuspended in PBS containing 0.05 mg/ml RNase A (Sigma), then incubated at room temperature for 30 min. After washing and staining with 10 mg/ml propidium iodide, we filtered the cells using a 60-μm mesh. At the last, 10,000 cells were analyzed by flow cytometry (FACSCalibur, BD Company) with ModFit software (Verity Software House, Inc.).

### Cell death analysis (7-aminoactinomycin D staining) and caspases activation

7-Amino-actinomycin D (7-AAD) was dissolved in acetone, diluted in PBS at a concentration of 200 μg/ml, and kept at 20°C in the dark until use. 100 μl 7-AAD solution was added to 2.5×10^6^ cells in 1 ml PBS and incubated for 20 min at 4°C without light. Cells were washed and total DNA was stained with 7-AAD (20 μl per sample). For assessing apoptosis, 5×10^5^ cells were resuspended in PBS and analyzed by flow cytometry. The total DNA content (stained by 7-AAD) was determined using CellQuest (Becton Dickinson) and FCS Express software (De Novo Software). Simultaneously, we measured caspase-3, caspase-8 and caspase-9 activity using Western blot by detecting their activated subunits.

### Reverse transcription and quantitative real-time PCR

RNA was isolated using the RNAprep Pure Cell/Bacteria Kit (TianGen, Beijing, China) according to the manufacturer's instructions and digested with DNase I to prevent amplification of genomic DNA. Reversed transcription was performed using QuantScript RT Kit (TianGen, Beijing, China) and gene expression analyzed using the iCycler iQ PCR detection system (Bio-Rad, Hercules, CA). The PCR amplification conditions were as follows: pre-denaturing at 95C for 3 min, followed by 40 cycles of amplifications by denaturing at 95°C for 30 s, annealing at 60°C for 1 min, extension at 72°C for 1 min. After a final extension at 72°C for 10 min, the amplified products were subjected to a stepwise increase in temperature from 55 to 95°C to construct dissociation curves. The following primers were used: cFLIP_L_ forward: 5′-AGTCCAGCCAAGAAGCAAGA-3′, cFLIP_L_ reverse: 5′-TGTAGCTCTCTTCATGTATG-3′; cFLIP_S_ forward: 5′-GTCCAGCCAAGAAGCAAGA-3′, cFLIP_S_ reverse: 5′-TTCTTTACCAAACACACGC-3′. GAPDH was used as a reference gene with the forward primer: 5′-ACAAGATGGTGAAGGTCGGTGTGA-3′ and reverse primer: 5′-AGCTTCCCATTCTCAGCCTTGACT-3′. Each sample was run and analyzed in triplicate.

### Western blot

At harvest, cells were removed using 0.25% Trypsin/EDTA (Invitrogen) for 3 min. Trypsinized cells were added to the media and centrifuged at 1,000 rpm at 4°C for 3 min. Pellets were washed with PBS and centrifuged again. Pellets were resuspended in 50 μl lysis buffer (10 mM Tris-HCl, pH 7.4, 150 mM NaCl, 1% Triton X-100, 1% SDS, 1 mM Na_3_VO_4_ and PMSF, 1 uM okadaic acid, and 10 μg/ml leupeptin, aprotinin, and pepstatin) and sonicated for 4 s at 40% followed by centrifugation in a microfuge at high speed for 15 min. BCA Assay Kit (Thermo Scientific, Rockford, USA) was used to determine the concentration of protein. Then, 30 μg of protein sample was electrophoresed on an 10% polyacrylamide SDS gel and transblotted onto a PVDF membrane under 250 mA for 90–150 min. The membranes with protein were incubated with primary and secondary antibody in turn. The hybridizing signals were analysized using ECL reagents (Amersham, GE Healthcare) in an automatic gel image analysis system (Tanon4500, Shanghai, China) according to the manufacturer's instructions.

### Animal model

Male Sprague-Dawley (SD) rats weighing 200–225 g were provided by Shanghai Laboratory Animal Center. All procedures abided by the Criteria for Care and Use of Laboratory Animals of Cangzhou Center Hospital, and were approved by the Ethics Committee for Experimental Research of Cangzhou Center Hospital. Diabetes was induced by a single intraperitoneal (i.p.) injection of 65 mg/kg body weight (BW) streptozotocin (STZ) in sodium citrate buffer (0.01 M, pH 4.0). Controls were injected with an equivalent amount of sodium citrate buffer alone. Only those rats with non-fasted plasma glucose concentrations >16.7 mM 1 week after STZ injection were recruited in the study. The rats were randomly distributed into the emodin (dissolved in corn oil)–treated and corn oil–treated (control) groups. The rats in the control and emodin group were administered emodin at 30 mg/kg/d or the same volume of corn oil, respectively, via oral gavage once daily for 10 consecutive days. This dose of emodin has been proved to be safe for SD rats [18].

### Measurement of proteinuria, blood glucose and glomerular filtration rate (GFR)

The level of proteinuria was determined from total urine volume of 24 h before sacrifice. The glucose level in the blood sample collected from the abdominal aorta was measured using blood glucose detection kit assays (Jiancheng Bioengineering Company, Nanjing, China). For GFR, rats were anesthetized by intraperitoneal injection of pentobarbital (50 mg/kg BW). Physiological saline solution (PSS) containing 10 mg/ml FITC-inulin was infused into the left jugular vein at a rate of 1 ml/h per 100 g BW. After a 1 h equilibration period, a blood sample (≈100 ml) was taken and urine was collected during the next 30-min period. Concentration of inulin was measured using a PerkinElmer 2030 Multilabel Reader (Victor X3, PerkinElmer Shelton, CT). FITC-inulin was measured with excitation at 485 nm and emission at 538 nm.

### Statistical analysis

Data were reported as means ± SE unless otherwise stated. The one-way ANOVA and Student *t* test were used to analyze the differences among/between groups, respectively. SPSS (v.19, IBM, USA) was used to assess date. P<0.05 was considered statistically significant.

## Results

### Emodin suppresses accelerated growth of MCs induced by HG

**Figure 1:**
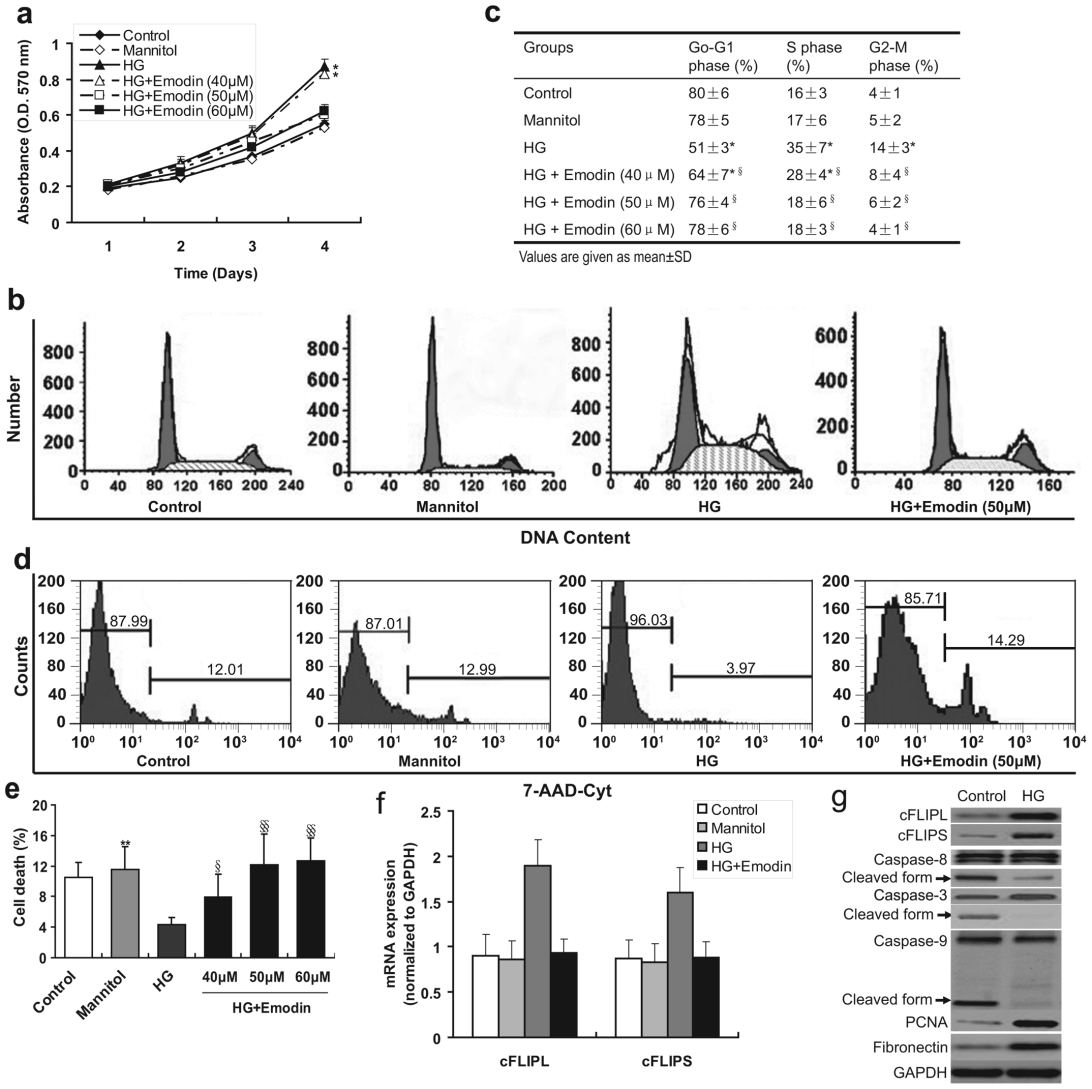
Emodin counteracts high glucose (HG)-induced growth in rat mesangial cells (MCs). (**a**) MTT assay shows HG but not mannitol promotes cell growth of high-passage MCs, the growth can be effectively arrested by 50 μM emodin. (**b, c**) Flow cytometry analysis of cell cycle by detecting propidium iodide staining reveals that the HG results in the decreased G0–G1 phase and increases S phase, however, it can be converted by 50 μM emodin. (**d, e**) Flow cytometry analysis of cell apoptosis by detecting 7-AAD staining shows fewer apoptotic cells in HG group. (f) mRNA expressions of cFLIP_L_ and cFLIP_S_ (normalized to GAPDH expression) were determined by quantitative real-time PCR, results showed that HG could induce elevated expression of both cFLIP_L_ and cFLIP_S_ (cFLIP_L/S_), and the elevated expression could be repressed by 50 μM emodin. (g) Western blotting reveals that HG medium promotes proliferation and Fibronectin synthesis, and inhibits apoptosis of MCs via blocking both extrinsic and intrinsic apoptosis pathway. *P<0.05 vs. control; **P<0.01 vs. control. ^§^P<0.05 vs. HG group; ^§§^P<0.01 vs. HG group.

The cell viability assay by MTT showed that HG could markedly increase the number of living MCs, which could be suppressed by emodin in a concentration-dependent manner (Fig. 1a). The MCs viability was not altered by emodin under normal glucose (Fig. 1a). The similar result was also observed by Li and his colleagues [19]. Cell viability between controlled groups was further determined by patterns of cell cycle distribution (Fig. 1b & 1c) and cell death analyses (Fig. 1d). Compared with control medium, HG induced a decrement of cell proportion in G0–G1 phase from 80% to 51% (*P*<0.05), increment in S phase from 16% to 35% (*P*<0.05) and G2-M phase from 4% to 14% (*P*<0.05) (Fig. 1c). However, the increased cell cycle was significantly suppressed by emodin in a concentration-dependent manner (*P*<0.05, Fig. 1c). Meanwhile, HG resulted in less cell death, which was also suppressed by emodin (Fig. 1e). These results suggested that emodin can suppress HG-induced cell viability through promoting apoptosis and restraining proliferation.

### cFLIP is necessary for HG-mediated proliferation

**Figure 2:**
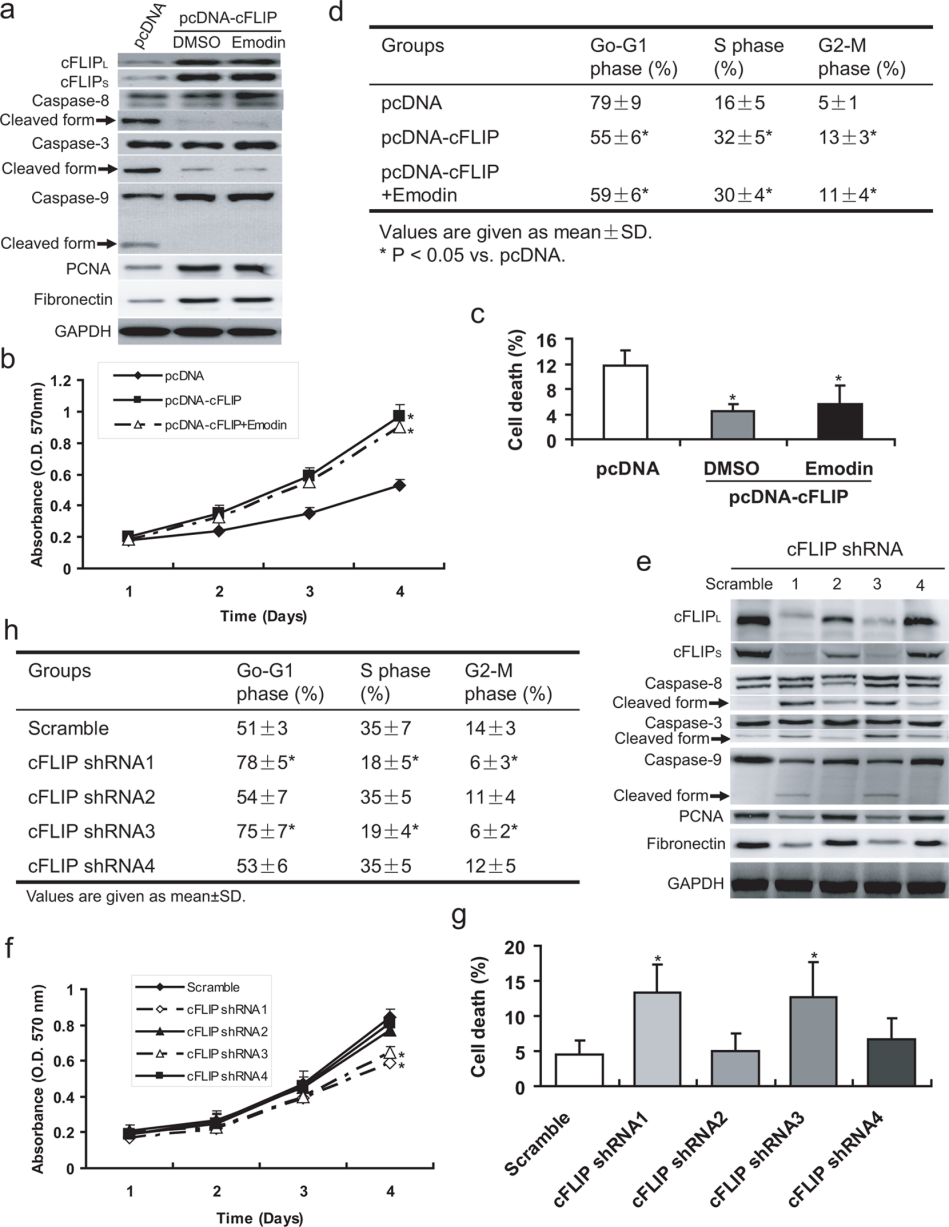
cFLIP is essential for HG-induced cell growth in rat MCs. (**a**) In normal-glucose condition, over-express of cFLIP promotes proliferation and Fibronectin synthesis, and inhibit apoptosis via blocking both extrinsic and intrinsic apoptosis pathway. The increased proliferation and Fibronectin synthesis can't be suppressed by by 50 μM emodin. (**b**) MTT assay shows over-express of cFLIP in normal-glucose medium can increase cell viability. (**c, d**) Flow cytometry analysis indicates that over-express of cFLIP in normal-glucose medium decrease G0–G1 phase and cell death, and increases S phase. (**e**) Western blotting reveals that knockdown of cFLIP using shRNA strategy can suppress proliferation and Fibronectin synthesis, and promote apoptosis via both extrinsic and intrinsic apoptosis pathway. (**f**) MTT assay shows knockdown of cFLIP decreases cell viability. (**g, h**) Flow cytometry analysis indicates that knockdown of cFLIP increases G0–G1 phase and cell death, and decreases S phase. *P<0.05 vs. scramble.

In HG medium, MCs expressed remarkably more cFLIP (Fig. 1f & Fig. 1g). So, it was thought to be necessary in HG-mediated proliferation. Then, a stable cell line overexpressing cFLIP was established (Fig. 2a). The cell viability assays suggested that MCs with overexpressed cFLIP (MC-cFLIP) underwent a process of increased proliferation and less apoptosis (Fig. 2a–d). Additionally, the process couldn't be repressed by Emodin (Fig. 2a–d). These results revealed that Emodin prevented proliferation and promoted apoptosis through inhibiting cFLIP. To test the cFLIP effects on HG-mediated proliferation, we also stably knocked down cFLIP (full-length) in MCs with short hairpin RNA (shRNA). Then, stably transfected cells were cultured in HG media. cFLIP expression was significantly decreased by shRNA1 and shRNA3 (Fig. 2e). Knockdown of cFLIP significantly decreased the number of viable cells assessed by MTT and proliferating cell nuclear antigen (PCNA) determined by western blotting (Fig. 2e & 2f). Silent cFLIP resulted in more cell death (*P*<0.05, Fig. 2g). Meanwhile, down-regulated cFLIP also remarkably decreased S and G2-M phase cells (*P*<0.05, Fig. 2h). Apoptosis can be initiated by both extrinsic and intrinsic pathway [20]. To test the pathway, through which the cFLP initiates apoptosis, caspase-8 (extrinsic signaling marker), caspase-3 (intrinsic signaling marker) and caspase-9 (intrinsic signaling marker) were simultaneously examined by western blotting, showing both pathways were involved in inhibited apoptosis caused by cFLIP (Fig. 2a & e). These results suggest that cFLIP is essential for HG activated proliferation and inhibited apoptosis.

### cFLIP is necessary for HG-mediated fibronectin expression

Extracellular matrix (ECM) protein produced by MCs was also believed to be correlated closely with deterioration of renal function in diabetic patients [21]. So, the fibronectin expression of MCs was evaluated. Results suggested that overexpressing of cFLIP can effectively result in fibronectin generation (Fig. 2a). Moreover, knockdown of cFLIP suppressed fibronectin induced by HG (Fig. 2e). These results suggested cFLIP could accelerate DN development via promoting MCs proliferation and ECM secretion.

### Emodin suppresses HG-mediated cFLIP generation in MCs

**Figure 3:**
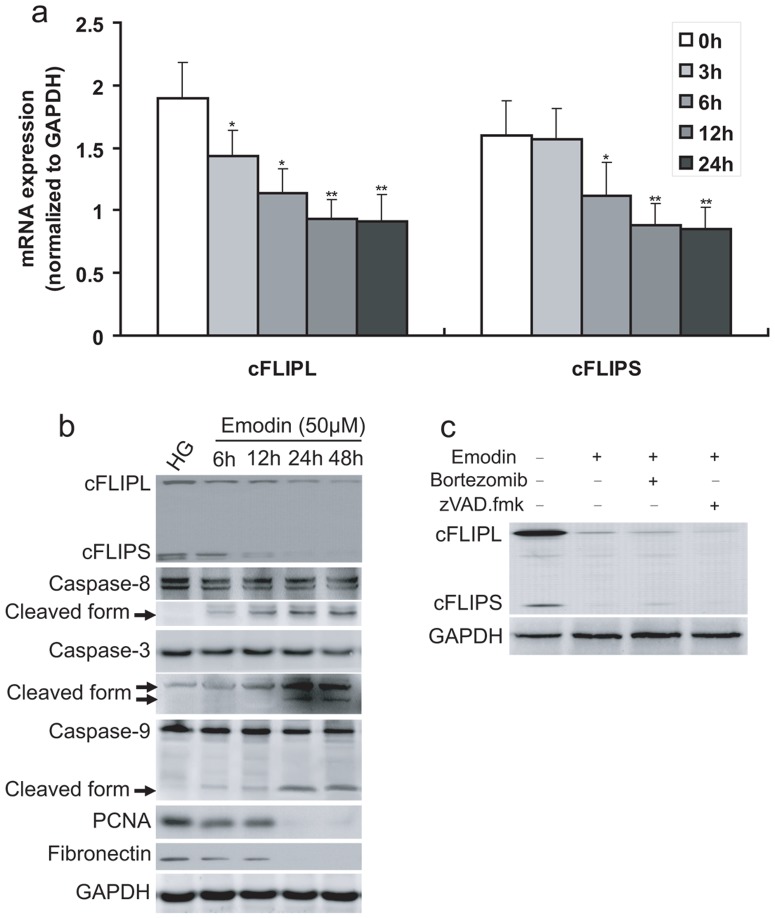
Emodin suppresses HG-induced cFLIP generation but not repressing its activation and increasing degration. (**a, b**) MCs were cultured in HG media and treated with 50 μM emodin for indicated times. Kinetic analyses of PCR and western blotting results show a time-dependent decrease of cFLIP_L/S_. (**c**) Cells were cultured in HG media and treated for 24 h with 50 μM emodin or plus 6 nM bortezomib or 50 mM zVAD.fmk (added 1 h before other substances). Expression of cFLIPL and cFLIPS was determined by western blotting. *P<0.05 vs. control; **P<0.01 vs. control. ^§^P<0.05 vs. HG group; ^§§^P<0.01 vs. HG group.

To test the effect of emodin on cFLIP generation, we examined cFLIP in MCs in both mRNA and protein level. Results revealed that emodin could suppress both cFLIP_L_ and cFLIP_S_ generation in transcriptional level (Fig. 2a & 3a). Then, kinetic analyses for effect of emodin on suppressing cFLIP were addressed in MCs, which was cultured in HG media. Results showed a time-dependent decrease of mRNA as well as protein levels of cFLIP_L_ and cFLIP_S_ by emodin (Fig. 3a & 3b). Moreover, time-dependent changes of caspase-3/8/9, PCNA and fibronectin induced by emodin were also observed (Fig. 3b). These results suggested that emodin suppresses HG induced proliferation through inhibiting cFLIP.

As previously reported, the observed decrease in cFLIP over time in the present of emodin may involve JNK-mediated proteasomal degradation of cFLIP [22]. To test whether emodin-mediated down-regulation of cFLIP involves proteasomal degradation or cleavage by caspases, we used the proteasome inhibitor bortezomib and the pan-caspase inhibitor zVAD.fmk. Result showed that degration of cFLIP protein was neither prevented by bortezomib nor by zVAD.fmk (Fig. 3c), supporting the notion that cFLIP is regulated by emodin at transcriptional level.

### Emodin decreases proteinuria and fibronectin expression in diabetic rats

**Figure 4:**
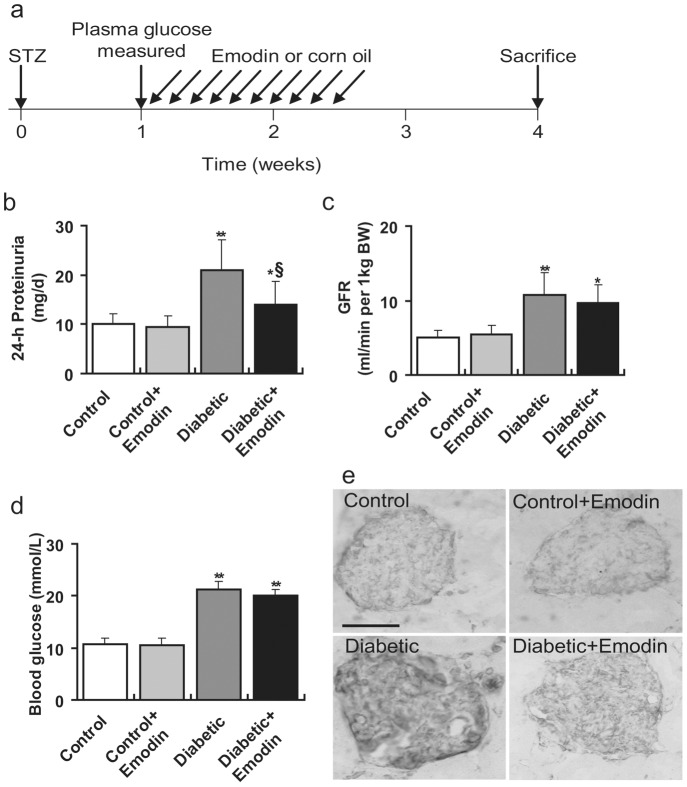
Emodin decreases proteinuria and fibronectin secretion in diabetic rats. (**a**) Schematic description of experiment as detailed in the methods section. (**b**) Proteinuria was determined from total urine volume of 24 h before sacrifice. Emodin can significantly decrease elevated proteinuria by diabetes. (**c, d**) GFR and blood glucose was measured 6–12 h before sacrifice. Emodin has no obvious effect on GFR and blood glucose diabetic rats. (**e**) fibronectin expressions were detected by immunohistochemistry staining of kidney sections from indicated groups. Emodin significantly represses fibronectin secretion in diabetic rats. Scale, 50 μm. *P<0.05 vs. control; **P<0.01 vs. control. ^§^P<0.05 vs. HG group.

As a definite index of renal function in early-stage of diabetic nephropathy [23], 24 h proteinuria was assessed 4 weeks after induction of diabetes. To determine the fate of diabetic rats, we delivered emodin as schematically illustrated in Fig. 4a. As shown in Fig. 4b, proteinuria is abnormally increased in diabetic rats from 10.1±2.1 mg/d to 21.0±3.1 mg/d (*P*<0.01; Fig. 4b). To measure the effect of emodin on GFR change, 30 mg/kg/d emodin was administrated to both control and diabetic groups for 10 consecutive days. In diabetic but not control groups, we observed significantly decreased proteinuria by emodin (Fig. 4b). The decrease of proteinuria in present of emodin may be caused by AMPK-mediated antidiabetic function and consequently decreased GFR [24]. So the GFR and blood glucose at the same time were also measured, showing that no significant change in GFR and blood glucose was found (Fig. 4c & 4d). At the last, the expression of fibronectin (another contributing factor for kidney function deteriorating) was determined by immunohistochemistry (IHC). As shown in Fig. 4e, diabetes gives rise to increased fibronectin that can be suppressed by emodin. These results suggest that emodin can prevent the diabetes-induced decline of kidney function at least in part by controlling cell viability and fibronectin secretion.

## Discussion

Emodin has been proposed as a potential agent for treatment of several proliferative diseases such as diabetic vascular complications [25], liver cirrhosis [26], and tumors [27]. Previous results have also indicated that emodin could suppress cell proliferation and fibronectin expression in rat mesangial cells cultured under high glucose [19]. However, the precise mechanism, and the efficacy and safety in vivo are still being investigated. Consistent with previous data, our study demonstrated that down-regulated cFLIP by emodin ameliorated diabetic renal dysfunction via suppressing MCs proliferation and fibronectin expression.

**Figure 5:**
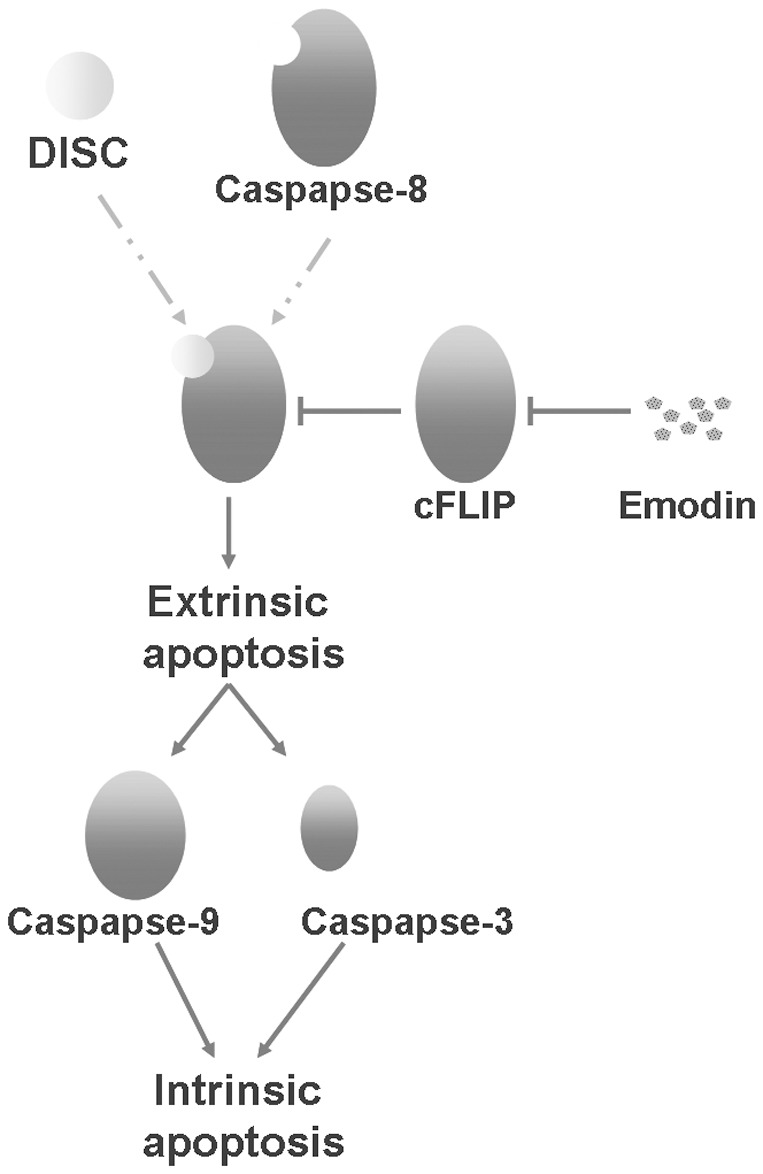
The function model of emodin in MCs illustrated by pathway map.

As recent studies, we approached aim to treat DN by preventing increased MCs viability and matrix protein secretion [28], [29] that are responsible for tissue homeostasis. Decreased in apoptosis and increased in proliferation both can contribute to elevated cell viabilities. Moreover, apoptosis can be induced by extrinsic and/or intrinsic pathway. Extrinsic apoptosis pathway is initiated by binding of death receptor ligands, such as TRAIL and CD95 ligand to their cognate death receptors at the membrane [30]. Receptor trimerization leads to intracellular recruitment of adaptor proteins such as Fas-associated death domain (FADD) which in turn enables activation and binding of caspase-8 to the death-inducing signaling complex (DISC) [31]. The intrinsic apoptosis pathway is started at the mitochondria which are permeabilized by intracellular death signals and release cytochrome c and other apoptogenic factors into the endochylema where caspase-9 and caspase-3 are activated [32]. Additionally, active caspase-8 can also activate caspase-3 resulting in apoptosis [30]. As a caspase-8 homolog lacking the catalytic cysteine, cFLIP is recruited to the same complexes, forms heterodimers with caspase-8, and blocks the formation of the proapoptotic caspase-8 homodimers [33]. Actived caspase-8 can truncate the BH3-only protein Bid into tBid which translocates to mitochondria to initiate mitochondrial outer membrane permeabilization [34], thereafter intrinsic pathway is initiated. A map abstract summarized this pathway was shown in Fig. 5. In our study, caspase-8, caspase-3 and caspase-9 were chosen as markers to measure the effect of emodin on apoptosis, showing that emodin could promote both extrinsic and intrinsic apoptosis in MCs.

For proliferation of MCs, cell cycle responded by flow cytometry and PCNA expression were assessed. Results showed that cell cycle of MCs can be effectively arrested by emodin. As a result, some cell functions such as ECM secretion were also suppressed. Our previous results demonstrated that emodin significantly suppressed HG-induced cell proliferation and protein expression of fibronectin via inhibiting the phosphorylation of p38MAPK and promoting Peroxisome Proliferator Activated Receptor-γ (PPARγ) protein expression [19]. And results from Marques-Fernandez revealed that cFLIP_L_ increased p38MAPK activation [35]. Thereby we assumed that cFLIP_L_ promoting cell proliferation and functions via p38MAPK activation. Fibronectin promoter contains a cAMP response element (CRE) located −170 bp of the gene [36]. Activated p38MAPK can result in the phosphorylation and activation of CRE binding protein (CREB) [37] that can bind with the CRE portion of the fibronectin gene, leading to fibronectin mRNA expression. In addition, activated p38MAPK could increase the protein expression of connective tissue growth factor (CTGF), which stimulated the cell proliferation and extracellular matrix accumulation [38].

cFLIP overexpression has been demonstrated to confer resistance to death receptor-mediated apoptosis as well as therapeutic drugs-mediated apoptosis [14], [39]. Evidences and corresponding molecular mechanisms of cFLIP to inhibit death receptor-mediated apoptosis in cancer cells and vascular smooth muscle cells have been well-established [14], [39], [40]. Consistent with the previous evidences, our data showed that cFLIP were essential for HG-mediated proliferation and ECM secretion of MCs. We also launched that emodin could effectively inhibit the generations of cFLIP_L_ and cFLIP_S_. This conclusion is based on findings that emodin caused reduced cFLIP mRNA levels, whereas proteasomal breakdown or caspase-mediated cleavage of cFLIP is not primarily involved.

In summary, our findings demonstrate that emodin alleviates progression of renal dysfunction in early-stage of STZ-induced diabetic Sprague-Dawley rats. *In vitro*, we introduce cFLIP as mechanisms contributing to the underlying effect. Through suppressing generation of cFLIP, emodin can promote apoptosis, and repress proliferation and EMC secretion of MCs. Inhibition of MC proliferation and EMC secretion may lead to suppression in mesangial hypertrophy, resulting in relief of eventual glomerulosclerosis and consequently deteriorating kidney function. Our study provides a cellular and molecular mechanism that likely contributes to the improvement of renal function by emodin in patients with DN.
